# Articular arthrodesis with the facet wedge technique for the treatment of unstable lumbar degenerative disease and associated conditions: A retrospective study of 96 patients

**DOI:** 10.1016/j.wnsx.2024.100351

**Published:** 2024-02-24

**Authors:** Guglielmo Iess, Daniel Levi, Raul Della Valle, Giulio Bonomo, Giovanni Broggi, Marcello Egidi

**Affiliations:** a Department of Neurosurgery, San Carlo Borromeo Hospital, Via Pio II, 3, Milan, Italy; b University of Milan, Via Festa del Perdono, 7, Milan, Italy; c Foundation IRCCS Carlo Besta Neurological Institute, Via Giovanni Celoria, 11, Milan, Italy; d Le Betulle Private Hospital, Viale Italia, 36, Appiano Gentile, Italy; e Piccole Figlie Hospital, Via Po, 1, Parma, Italy; f Fondazione I.E.N., Corso Venezia, 18, Milan, Italy

**Keywords:** Facet wedge, Articular arthrodesis, Lumbar instability, Lumbar degenerative disease, Synovial cyst, Adjacent segment disease

## Abstract

**Background:**

Lumbar articular fusion with the facet wedge (FW) technique is gaining increasing interest among surgeons for the treatment of vertebral instability due to its limited invasiveness and ease of use. Studies on cadavers have reported biomechanical properties similar to pedicle screws. Yet, the evidence supporting their use is still limited and moreover focused only on spinal degenerative disease.

**Methods:**

96 cases of lumbar articular fusion with the FW techniques performed at 3 different centers between 2014 and 2022 were retrospectively analyzed based on the specific surgical indications: 1) degenerative spondylolisthesis/unstable lumbar stenosis; 2) synovial cysts; 3) adjacent segment disease (ASD). Medical records were reviewed to identify rates of complications and measures of functional outcome (ODI, low back pain VAS and modified Macnab scale) were collected both at baseline and at the follow-up visits. Wilcoxon signed-rank test was adopted to test for significant functional improvements.

**Results:**

Significative clinical improvements were observed from baseline to follow-up regarding ODI and VAS scores. Overall rate of moderate and severe complications (according to Landriel–Ibañez scale) was 7.9%. Only 3.4% of patients with degenerative disease developed ASD requiring reoperations. Only one case of radicular deficit and one of device mobilization were reported. 2/4 cases of synovial cysts treated with unilateral fusions developed contralateral complications. 9 out of 16 (56.25%) patients who underwent long-term postoperative CT scans presented adequate degree of articular fusion.

**Conclusion:**

FW technique is easy, safe, and effective. Its low rate of complications justifies its use for cases of mild lumbar instability.

## 1 Introduction

Since the first description of a case of spinal fusion by Dr. Russell A. Hibbs in 1911, lumbar fusion has become one of the most routinely performed neurosurgical procedures. Despite being initially conceived to treat spinal deformities, nowadays lumbar fusion is used for a variety of different spinal conditions including spondylolisthesis, degenerative conditions of the spine, traumatic injuries, synovial cysts, scoliosis, spinal instability determined by primary and secondary tumors, spondylodiscitis, and adjacent segment disease (ASD).^1^ Depending on the specific vertebral elements subject to fusion, different methods of lumbar arthrodesis have been developed over time. Nowadays, the two mainstream techniques are the lumbar interbody fusion (LIF), which may be performed with different approaches including the posterior, anterior, transforaminal, lateral and oblique) and the posterolateral fusion (PLF).^1^ Although standalone arthrodesis has been widely used in the past, most surgeons now advocate the addition of spinal instrumentation in order to accelerate and increase fusion's rates.^2^^,^^3^

Pedicle screw fixation is considered the gold standard for instrumented fusion. Nevertheless, this procedure, apart from being technically quite demanding and lengthy, is associated with rates of screw misplacements ranging from 0 to 42% in literature.^4^ Although fortunately serious complications (i.e., nerve root injury, vascular or visceral perforations) are uncommon (0.6–11%), this technique may pose the vertebrae at risk for superior facet breach, which is known to increase instability and consequently to promote degenerative changes in the upper segment, leading to ASD eventually requiring a revision surgery.^5^

An alternative to classic LIF and PLF with pedicle screws instrumentation is represented by the facet articular fixation and fusion. Although for this purpose various techniques have been described (e.g., the transfacet screws described by Boucher, translaminar screws, interference screws) with the first case of facet fusion dating back to the year 1948, it has remained largely underused over time due to the common belief that it offers a minor degree of stability compared to transpedicular systems.^6^ Nevertheless, the concept of blocking the articular joints to stabilize a motion segment has always attracted many surgeons because of the potential for less morbidity due to its favorable ease of access, the limited extent of muscle division required compared to PLF, the limited (or non-existent) need for root and/or dural sac retraction and because it achieves fusion where the distance between the two vertebrae is minimal (limiting the amount of bone harvesting required for bone graft).^7^, ^8^, ^9^

Recently, a new method combining different concepts of facet articular fixation and fusion represented by the so-called “Facet Wedge” technique has been developed.^7^^,^^8^^,^^10^ This system has been tested in biomechanical studies on cadavers, reporting properties comparable to pedicle screws with regards to lateral bending, flexion, extension and even slightly superior for axial rotation.^10^ These characteristics were observed comparing the different fixation systems utilized both as stand-alone and also in association with LIF.^10^

In this article we describe the experience with the use of this novel device at three institutions for the treatment of three spinal lumbar conditions: 1) degenerative spondylolisthesis Meyerding type I/unstable lumbar stenosis; 2) lumbar synovial cysts; 3) ASD.

The primary objective of this study was to analyze the safety and efficacy profiles of this technique in a cohort of 96 patients.

## Consent

Institutional review board approval was obtained for this retrospective study. An informed consent waiver for each patient was granted.

## 2 Material and methods

In this retrospective study, a thorough research was conducted in the electronic medical records of the three institutions (ASST Santi Paolo e Carlo, Milan; Piccole Figlie Hospital, Parma; Private Hospital Le Betulle, Como) to identify cases in which the facet wedge system (DePuy Synthes, Raynham, Massachusetts, United States) was utilized during lumbar surgery from January 2014 to June 2022. The research retrieved 98 results (46 females and 52 males) of patients who were deemed as ideal candidates to undergo lumbar facet articular fixation and fusion with the facet wedge system, 96 of which were actually implanted, while in two patients (2.04%) FW intraoperative placement was abandoned and therefore facet fusion was not performed: in one case of mild degenerative spondylolisthesis (grade I) the bone quality of the articular processes was considered too inconsistent to allow for adequate screw insertion, while in another case of lumbar synovial cyst, spontaneous joint fusion was observed intraoperatively and therefore facet fixation and fusion was deemed as unnecessary. These patients were consequently excluded from the statistical analysis which therefore included 96 patients.

Before undergoing surgery, all patients suffered from either neurogenic claudication or chronic back pain, variably associated with signs of radiculopathy and attempted a trial of unsuccessful conservative treatment (such as non-steroidal anti-inflammatory drugs, corticosteroids, physical therapy) for at least 3 months. Radiological work-up always included lumbar Magnetic Resonance Imaging (MRI) and flexion-extension dynamic X‐rays. For the early cases involving FW implants, preoperative CT scans were also performed to assess facet joint orientation, shape, and potential obstructive tissues (e.g., osteophytes). However, as the experience of the surgical team with the device increased, lumbar MRI demonstrated of comparable utility in evaluating these characteristics. Consequently, the routine use of preoperative CT scans was discontinued.

Exclusion criteria for the implantation of the facet wedge system comprised spondylolisthesis with isthmic spondylolysis, proximal junctional kyphosis (defined as a proximal junctional sagittal Cobb angle greater than 10° and at least 10° > than the measurement before the previous lumbar fusion procedure) history of lumbar vertebral fractures, spinal metastases and severe osteoporosis (which represents a general contraindication to spinal instrumentation).

Before the operations, all individuals provided written informed consent, and all surgeries were performed according to the principles of the Declaration of Helsinki. Concomitantly with articular fusion, decompressive laminectomies, foraminotomies and microdiscectomies were variably performed on a case-by-case basis. In 31 cases post-operative lumbar MRIs were performed to assess spinal canal decompression.

Patients were generally advised to ambulate wearing a corset brace the day following surgery, and corset brace use was recommended for 4 weeks.

**Fig. 1:**
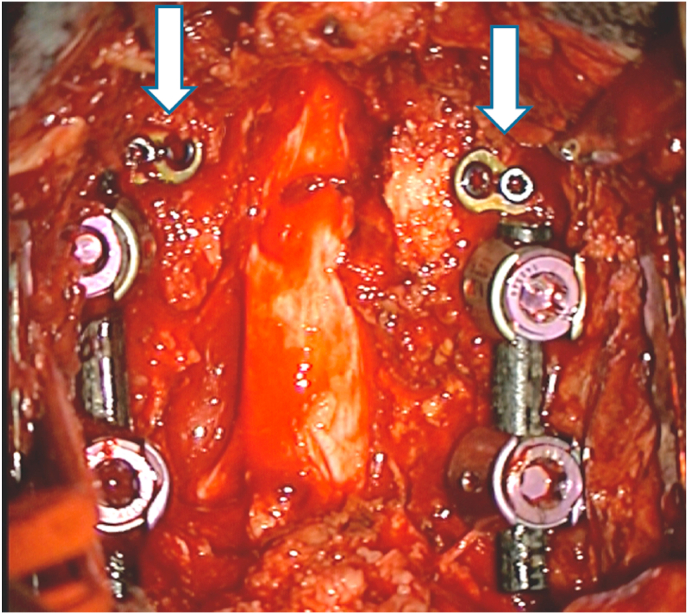
Intraoperative image illustrating the addition of the facet wedge device (white arrows) to the cephalad adjacent level for the treatment of ASD secondary to a previous LIF.

Patients were divided into three groups based on the specific surgical indications analyzed in our series, namely: 1) Either Meyerding grade I degenerative spondylolisthesis (DS) or unstable lumbar stenosis (ULS), defining instability as a vertebral translation >4 mm on motion radiographs (referred to in this text as the “DS/ULS group”; *n* = 81); 2) Synovial cysts (*n* = 8); 3) Late onset of ASD at the level cephalad to a previous LIF (i.e., as a treatment for ASD; Fig. 1) or initial signs of upper-level adjacent segment degeneration (ASDeg) at the time of the LIF procedure (i.e., as a prophylactic measure to prevent ASD) (referred to in this text as the “ASD group”; *n* = 7).

In this series the data we searched for in the medical records (charts, admission and discharge letters, follow-up visits) included: age, sex, surgical indication, ASA score, BMI, levels of fusion (e.g., L4-L5), length of hospital stay, revision surgeries for adjacent level disease or other causes, and postoperative complications (both medical and surgical). Surgical postoperative complications included: cerebrospinal fluid leaks, wound dehiscence, post-surgical hematoma requiring surgical drainage, spinal infections, radicular post-decompressive deficits, hardware failure (device mobilization or rupture) and ASD requiring reoperation. Medical complications (defined as occurring within 3 months from the operation) examined were pneumonia, deep vein thrombosis, strokes, urinary tract infections and cardiological complications.

Patient outcomes were assessed both preoperatively and at the final follow-up visits (which were performed between April 2022 and December 2022 by three independent investigators) by means of the Visual Analog Scale (VAS) to grade back pain, the Oswestry Disability Index (ODI) and the modified Macnab Scale.^11^ Five patients required reoperation for long-term complications (i.e., occurring after 6 months) and their clinical outcomes were assessed immediately before the revision surgeries. The Minimal Clinically Important Difference (MCID) of both ODI and VAS were calculated using an anchor-based method which exploited the Δ mean difference between preoperative and postoperative scores among patients who reported a small clinical improvement with the modified MacNab scale (i.e., equivalent to a “fair” outcome).

The long-term facet fusion (evaluated >6 months after the surgical procedure) was assessed in 16 patients, with 11 individuals undergoing lumbar CT scans for lumbar-related indications (e.g., recurrence of pain, onset of postoperative complications), while another 5 underwent abdominal CT scans at the same institutions for unrelated abdominal conditions. The decision to utilize both lumbar and abdominal CT images for facet fusion evaluation was based on studies indicating that, despite limitations (such as assessing the spinal cord and bone marrow), contemporary CT scanners with advanced methods of image reconstruction can accurately evaluate the lumbar spine in abdominal CT studies.^12^ The ordinal grading system by Miyashita et al was employed to assess the grade of fusion: Grade I denoted complete bone continuity enveloping the entire facet joint; Grade II indicated continuous but incomplete bone connection between facets; Grade III signified uncertain bony continuity between facets, and Grade IV indicated obvious nonunion.^13^ Adequate fusion was defined as Grade I or II fusion in one or both facet joints, as defined by the grading system by Miyashita et el. The ratings were conducted by two independent investigators, both of whom were unaware of the outcome data. Good intra- and inter-reader reliability was achieved, with a kappa value exceeding 0.70.

Mean follow-up time was 3 years and 9 months. 5 patients (5.21%) were lost to follow-up and other 2 patients (2.08%) died of other causes. Therefore, measures of functional outcome of these individuals were not available, and their follow-up coincided with the last medical record available for review.

### 2.1 Surgical technique

**Fig. 2:**
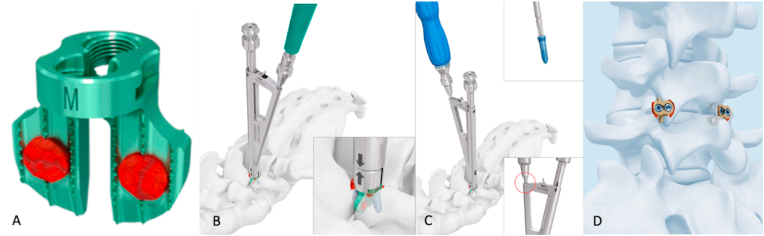
Illustrated schematically is the FW implant and its insertion technique. The FW (depicted in image a) is a titanium wedge-shaped intraarticular spacer with two perforations filled with bone grafts. It is equipped with two horizontal wings, allowing attachment to the medial and lateral facet processes using a pair of diagonal screws. Loading is facilitated by a triangular implant holder featuring a lateral guide, through which an awl and, subsequently, a diagonal screw are inserted into the articular processes (shown in images b and c). Following this, the holder is removed (image d), leaving the FW in place (Permission obtained from DePuy Synthes).

The day of surgery, the patient is positioned prone on a radiolucent table. Fluoroscopy is utilized to assess the correct level. After a midline skin incision and dissecting laterally the paravertebral musculature, the facet joints are exposed. The articular capsule is incised to expose the joint cavity and any additional osteophytes are removed using rongeurs or a bone drill. After removing the cartilage by applying manipulations with a rasp or with the aid of a monopolar coagulator, a facet wedge implant of adequate size (small, medium or large) to fit the articular space is selected and loaded on an implant holder (Fig. 2). These implants are titanium wedge-shaped devices that house perforations which are firmly packed with bone graft (obtained from the spinous process or the laminae of the patient) or bone inducing material to achieve joint fusion. Attention is paid to fill the implant until the bone (or bone graft substitute) protrudes from its perforations to ensure contact with the facet joint surfaces. The device is then introduced into the joint space with the aid of the implant holder. The facet wedge system is also provided with two shoulders from which two self-locking divergent screws are introduced into the bone of the two joint processes to stabilize the implant and obtain articular fixation. A final fluoroscopic control is performed at the end of the procedure to confirm proper hardware's placement.

In cases where laminectomies, foraminotomies or discectomies are also planned, facet wedge insertion is always carried out before performing such procedures to avoid the risk of weakening the superior articular process which would hamper articular arthrodesis.

### 2.2 Statistical analysis

Mean and Standard Deviation (SD) were used to describe continuous variables, while frequencies and percentages were utilized for categorical variables. We tested continuous variables for normality with the Shapiro–Wilk test and obtained statistically significant values for preoperative, postoperative (measured at the follow-up evaluation) and differential ODI and VAS scores. Therefore, non-parametric tests were used to compare these variables among different groups such as the Mann–Whitney U and Kruskal–Wallis tests for unpaired groups, and the Wilcoxon signed-rank test for paired comparative analysis. Fisher's exact test was used to compare frequencies among different groups.

Multiple logistic regression analysis was employed to predict the probability of achieving a MCID for ODI. The predictor variables used were ASA score, age, gender, BMI and surgical indications. The fitted model was confronted with an intercept-only model, moreover, resulting in a significant improvement. Moreover, by calculating the Variance Inflation Factors (VIF) between all single independent variables, we excluded possible multicollinearity issues. Spearman's rho was employed to investigate the correlation between follow-up time points and improvements in ODI and VAS among patients treated for DS/ULS (the only sizable sample suitable for this analysis). Additionally, ordinal logistic regression was chosen to analyze whether follow-up time (categorized into four specific lengths) predicted clinical outcomes, as described by the modified MacNab categories. We also utilized logistic regression to test whether the preoperative ASA score had any significative predictive effect on the onset of postoperative complications.

All *p*-values reported are two-tailed and a *p* < 0.05 was considered statistically significant.

Calculations and graphs were made using SPSS (IBM Corp. 2020 Release, IBM SPSS Statistics for MacOs, Version 26.0).

## 3 RESULTS

In our study mean patient age and BMI were 69.89 (SD ± 9.87) years and 27.68 (SD ± 6.92) respectively. 6 patients (6.3%) were operated with an ASA score of 1, 36 (37.5%) with an ASA score 2 and the remaining 54 (56.3%) had an ASA score of 3. Full descriptive statistics are illustrated in Table 1.

**Table 1:** Table illustrating descriptive statistics of the study. FW, facet wedge; BMI, body mass index; SD, standard deviation; ASA, American society of anesthesiologists. DS/ULS, degenerative spondylolisthesis/unstable lumbar stenosis; LSC, Lumbar synovial cysts; ASD, adjacent segment disease.

Parameter	
**Age, years [mean ± SD]**	69.89 ± 9.87
**Gender, n [%]**	Females = 45 [46.9%]; Males = 51 [53.1%]
**Follow-up, months [mean ± SD]**	DS/ULS = 49.21 ± 28.99; LSC = 42 ± 26.90; ASD = 47.5 ± 20.47
**BMI [mean ± SD]**	27.68 ± 6.92
**ASA, n [%]**	ASA 1 = 6 [6.3%]; ASA 2 = 36 [37.5%]; ASA 3 = 54 [56.3%]
**Levels fused with FW, n [%]**	L1-L2 = 1 [3%]; L2-L3 = 7 [7.3%]; L3-L4 = 35 [36.5%]; L4-L5 = 42 [43.8%]; multilevel = 11 [11.5%]
**Hospital stay, days [mean ± SD]**	5.90 ± 5.31

Statistically significant improvements were observed in ODI scores across all three surgical categories, with mean changes ranging from 26.90 ± 19.84 in DS/ULS to 31.29 ± 25.91 in LSC, from baseline to follow-up assessments (Table 2). Likewise, VAS scores for low back pain demonstrated statistical improvements across the three categories, varying from 4 ± 3.65 in the ASD group to 5 ± 2.38 in the LSC group (Table 3). Detailed Modified MacNab scores are also provided in Table 4. The three different indications for facet wedge fusion were similar with regards to functional improvement as described by ODI score (*U* = 1.146, *p* = 0.564, Kruskal–Wallis test), Macnab scale (*U* = 0.510, *p* = 0.775, Kruskal–Wallis test) and VAS low back pain scores (*U* = 0.470, *p* = 0.790, Kruskal–Wallis test).

**Table 2:** Table illustrating the distributions of the mean preoperative and postoperative (including their difference) ODI scores throughout the three different indications for articular fusion with facet wedge. ODI, Oswestry disability index; DS/ULS, degenerative spondylolisthesis/unstable lumbar stenosis; LSC, lumbar synovial cysts; ASD, adjacent segment disease.

Indication	Preoperative ODI (mean ± SD)	ODI at last follow-up (mean ± SD)	Difference	p^a^
**DS/ULS**	52.84 ± 11.90	25.93 ± 16	26.90 ± 19.84	<0.001
**LSC**	51 ± 19.74	19.71 ± 22.25	31.29 ± 25.91	0.017
**ASD**	54.29 ± 14.59	19.57 ± 17.20	27.71 ± 27.02	0.043

a Wilcoxon Signed Ranked Test.

**Table 3:** Table illustrating the distributions of the mean preoperative and postoperative (including their difference) back pain VAS scores throughout the three different indications for articular fusion with facet wedge. VAS, visual analog scale; DS/ULS, degenerative spondylolisthesis/unstable lumbar stenosis; LSC, lumbar synovial cysts; ASD, adjacent segment disease.

Indication	Preoperative VAS (mean ± SD)	VAS at last follow-up (mean ± SD)	Difference	p^a^
**DS/ULS**	7.91 ± 1.61	3.12 ± 1.9	4.79 ± 2.4	<0.001
**LSC**	7.71 ± 2.43	2.71 ± 3.04	5 ± 2.38	0.012
**ASD**	7.57 ± 2.07	3.57 ± 2.76	4 ± 3.65	0.034

a Wilcoxon Signed Ranked Test.

**Table 4:** Table illustrating the distribution (in %) of the different outcomes (as described by the modified Macnab scale) throughout the three different indications for articular fusion with facet wedge. DS/ULS, degenerative spondylolisthesis/unstable lumbar stenosis; LSC, lumbar synovial cysts; ASD, adjacent segment disease.

Indication	Modified Macnab classification of postoperative outcome (%)
**DS/ULS**	17.3% excellent, 62.7% good, 13.3% fair, 6.7% poor
**LSC**	25% excellent, 50% good, 0% fair, 25% poor
**ASD**	14.3% excellent, 57.1% good, 0% fair, 28.6% poor

The MCID anchor-based method yielded absolute cutoff values of ≃ 12 (11.8) and ≃ 3 (2.9) points for ODI and VAS respectively. In the group of patients treated for DS/ULS, 93.2% (69/74) and 87.8% (65/74) reached the MCID for ODI and VAS respectively, while in the other two groups with smaller samples the percentages were lower as described in Table 5.

**Table 5:** Table illustrating the proportion of patients achieving a MCID for both ODI and low back pain VAS scores among patients treated for the three different indications for articular fusion with facet wedge. MCID, minimal clinically important difference; ODI, Oswestry disability index; VAS, Visual analog scale; DS/ULS, degenerative spondylolisthesis/unstable lumbar stenosis; LSC, lumbar synovial cysts; ASD, adjacent segment disease.

Indication	MCID ODI, % (n/total)	MCID VAS, % (n/total)
**DS/ULS**	93.2% (69/74)	87.8% (65/74)
**LSC**	75% (6/8)	75% (6/8)
**ASD**	71.4% (5/7)	57.1% (4/7)

**Table 6:** Distribution of surgical and medical complications among the three different surgical indications. DS/ULS, degenerative spondylolisthesis/unstable lumbar stenosis; LSC, lumbar synovial cysts; CSF, cerebrospinal fluid; ASD, adjacent segment disease; DVT, deep vein thrombosis; UTI, urinary tract infections.

Complications	n. (%)
DS/ULS (*n* = 81)	
*Surgical complications*	
Dural tears without persistent CSF leaks	4 (4.9%)
ASD	3 (3.7%)
Dural tears with persistent CSF leaks	1 (1.2%)
Device mobilization	1 (1.2%)
Radicular deficit	1 (1.2%)
*Medical complications*	
Stroke	1 (1.2%)
DVT	1 (1.2%)
Atrial fibrillation	1 (1.2%)
UTI	1 (1.2%)
	TOTAL = 14 (17.3%)
**LSC (n = 8)**	
*Surgical complications*	
Controlateral LSC	1 (12.5%)
Controlateral disc herniation	1 (12.5%)
	TOTAL = 2 (25%)
**ASD (n = 7)**	
UTI	1 (14.3%)
	TOTAL = 1 (14.3%)

By employing multiple logistic regression analyses, none of the five different parameters tested (ASA score, age, BMI, gender, and surgical indication) emerged as a significant negative predictor of achieving an MCID for ODI (see Table 6). However, it is worth noting that the ASA score approached significance (*p* = 0.075; Table 7).

**Table 7:** Multiple logistic regression illustrating the effects of various clinical and surgical predictor variables on the probability of achieving a MCID for ODI after articular fusion with the facet wedge system. Surgical indications include degenerative spondylolisthesis type I/unstable lumbar stenosis (DS/ULS), lumbar synovial cysts (LSC) and adjacent segment disease (ASD). *p* values which are statistically significant are evidenced in bold. OR, odds ratio; BMI, body mass index; ASA, American society of anesthesiologists.

Clinical parameter	OR	95% CI for OR	p
**ASA**	0.229	0.045, 1.157	0.075
**BMI**	0.879	0.708, 1.092	0.245
**Age**	0.927	0.825, 1.043	0.209
**Gender**	3.449	0.668, 17.808	0.139
**Surgical indication**			
**DS/ULS**^a^			
**LSC**	7.084	0.718, 69.866	0.094
**ASD**	0.586	0.044, 7.876	0.687

a Set as the reference category.

**Fig. 3:**
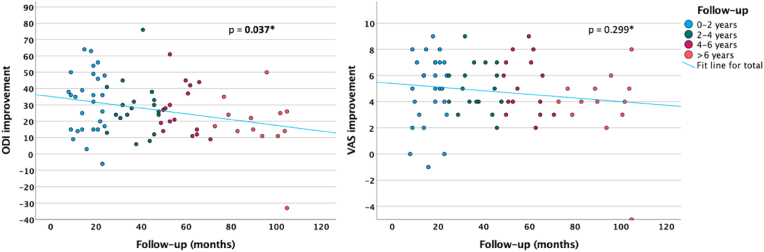
Scatterplots illustrating the correlation between follow-up time points and improvements in ODI and low back pain VAS scores, respectively, among patients treated for DS/ULS (*n* = 74). Significant correlations are highlighted in bold. ODI, Oswestry disability index; VAS, Visual analog scale; DS/ULS, degenerative spondylolisthesis/unstable lumbar stenosis. *Spearman's rho correlation.

**Fig. 4:**
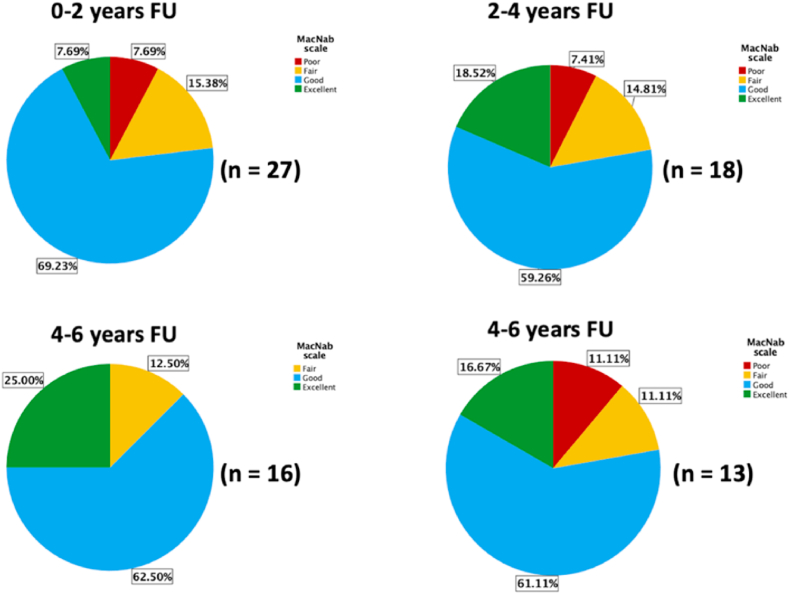
Pie charts illustrating the distributions (in %) of modified MacNab scale outcomes among patients treated for DS/ULS, categorized based on different follow-up intervals. DS/ULS, degenerative spondylolisthesis/unstable lumbar stenosis; FU, follow-up.

We observed a weak but statistically significant correlation between follow-up time points and ODI improvements (*ρ* = −0.243, *p* = 0.037) when considering only the DS/ULS group (the sole cohort with a statistically acceptable sample for this analysis), suggesting degenerative disease progression. However, no such association was observed for VAS scores for low back pain (*ρ* = −0.122, *p* = 0.299; Fig. 3). Grouping DS/ULS patients into four categories based on their follow-ups (Group 1: 0–2 years; Group 2: 2–4 years; Group 3: 4–6 years; Group 4: >6 years) revealed no significant difference in terms of MacNab scale outcomes (Fig. 4) between the four categories (*B* = −0.013; 95% CI -0.422, 0.395; *p* = 0.949; simple ordinal logistic regression).

Regarding lumbar fusion rates, out of the 16 analyzed CT scans, we identified 3 cases (18.8%) with grade I fusion, 6 cases (37.5%) with grade II, and 7 cases (43.8%) with grade III. No cases of grade IV (complete nonunion) were reported. Therefore, 56.2% of the patients undergoing CT scans achieved adequate fusion vs. 43.8% who did not. No significant difference in clinical outcomes, as expressed by the MCID, was noted among the patients with adequate fusion vs those with no solid evidence of arthrodesis for both ODI and VAS (88.9% achieving the MCID and 11.1% not in the group of individuals with adequate fusion vs 57.1% achieving the MCID and 42.9% not in the non-fusion group; *p* = 0.262; Fisher's exact test). Moreover, no evidence of device mobilization was observed in this subset of patients.

Concerning postoperative complications (Table 7), three patients (3.7%) in the DS/ULS group treated with standalone FW developed ASD at the upper level which presented in all three cases with discal herniations determining radiculopathy and back pain. Rates of ASD were not different among patients undergoing multilevel articular facet wedge fusion vs. those operated of a single level fusion (*p* = 0.772; Fisher's exact test). Conversely, none of the seven patients in the ASD group underwent revision surgery due to surgical complications (mean follow-up of this subset of patients = 3 years and 6 months). Five intraoperative dural tears in the DS/ULS group (6.17%) were reported during posterior spinal decompressions which were either repaired with direct suture or sealed with fibrin glue depending on the extension of the laceration, and subsequently managed with 1 or two days of bed rest. Nevertheless, only one of these 5 patients (1.23%) developed a persistent postoperative CSF leak which was successfully treated only after placement of an external lumbar drain which was held for a few days. The same patient also required revision surgery because of a facet wedge migration which was noted on a spinal MRI.

**Fig. 5:**
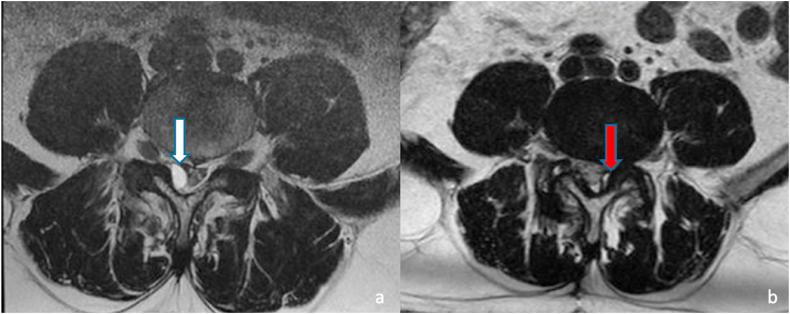
Axial T2-weighted MRI images illustrating the case of a right lumbar synovial cyst treated (white arrow, figure a on the left) with excision and monoarticular ipsilateral fusion with FW technique in 2014. Five years later the patient developed a contralateral cyst (red arrow, figure b on the right) which required revision surgery. (For interpretation of the references to colour in this figure legend, the reader is referred to the web version of this article.)

Two patients out of 8 in the synovial cyst group required revision surgeries (25%). Of note, both patients had single-levels, ipsilateral articular fusions with FW. The first was a 51-years old man who developed a contralateral synovial cyst at the same level 5 years later and therefore underwent revision surgery with removal of the cyst and articular fusion of the other articular joint (Fig. 5). After the revision surgery, the patient enjoyed a good outcome, with a decrease in ODI score from 26 to 2 and a reduction of VAS score for low back pain from 4 to 0. The second similar case we describe, is the one of a patient who underwent a L3-L4 synovial cyst excision and concomitant ipsilateral FW fixation who developed a contralateral disc herniation at the same spinal level one year later. Similarly, to the other case, the patient had a good outcome after the revision surgery. Of the 4 patients with synovial cysts who underwent unilateral FW implantations 2/4 (50%) required revision surgeries compared to the 4 subjects treated with bilateral implantations among whom no reinterventions were needed.

No episodes of spondylodiscitis, postoperative symptomatic hematomas requiring surgical drainage and wound dehiscence were reported. None of the clinical and surgical parameters analyzed in our study (ASA score, age, BMI, gender, and surgical indication) emerged as a significant predictor of the onset of postoperative complications (both medical and surgical) utilizing multiple logistic regression analysis (Table 8).

**Table 8:** Multiple logistic regression illustrating the effects of different clinical and surgical predictor variables on the probability of suffering from a medical or surgical postoperative complication. Surgical indications include degenerative spondylolisthesis type I/unstable lumbar stenosis (DS/ULS), lumbar synovial cysts (LSC) and adjacent segment disease (ASD). *p* values which are statistically significant are evidenced in bold. OR, odds ratio; BMI, body mass index; ASA, American society of anesthesiologists.

Clinical parameter	OR	95% CI for OR	p
**ASA**	0.722	0.336, 1.551	0.404
**BMI**	0.883	0.751, 1.038	0.130
**Age (years)**	1.009	0.953, 1.069	0.754
**Gender**^b^	1.159	0.380, 3.535	0.795
**Surgical indication**			
**DS/ULS**^a^			
**LSC**	1.566	0.172, 14.236	0.690
**ASD**	1.088	0.052, 22.657	0.957

a Set as the reference category.

b 0 = females; 1 = males.

## 4 Discussion

Facet wedge is an emerging technique potentially combining both articular fixation and arthrodesis at the same time.^10^ The ease of access to the facet joints, the limited extent of muscle retraction compared to posterolateral fusion (especially when the mini-open percutaneous technique is used), its rapid implantation and the unneeded retraction or potential injury of the neural elements make it an attractive option for minimally invasive lumbar fusion. Nevertheless, although several studies have reported FW fusion to be a safe and effective method, the body of literature describing their use is still limited, especially for what concerns long-term data.^7^^,^^8^^,^^13^

Interestingly, while the other articles in literature have reported the use of this technique associated with canal decompression in lumbar spinal stenosis with signs of mild instability, to our knowledge this is the first report describing its application for two novel indications, namely for lumbar synovial cysts and for the treatment/prevention of ASD.^8^^,^^14^

Because these two indications represent only a small percentage of our cohort of patients, for the purpose of scientific rigor the discussion which follows will focus on the conditions analyzed separately. Nevertheless, all surgical indications reported similar improvements after surgery in the three outcome measures tested (ODI, modified Macnab scales and VAS for low back pain). Unlike other reports found in the literature on LIF and posterolateral fusion, which identified significant associations between certain preoperative risk factors such as higher BMI and older age with worse clinical outcomes, none of the preoperative variables (Age, ASA, BMI, gender, and surgical indication) appeared to be significant predictors.^15^, ^16^, ^17^ However, it is noteworthy that ASA approached significance.

Similarly to other series describing the use of FW for the treatment of lumbar spinal stenosis, our patients with this condition reported significant improvements with regards to ODI (26.90 ± 19.84) and VAS for low back pain (4.79 ± 2.4), and moreover, 17.3% had excellent results, 62.7% good, 13.3% fair and only 6.7% poor. These results were sustained over time, as demonstrates the mean follow-up of more than 4 years (49 ± 28 months) of this category.^8^^,^^14^ Moreover, 3.1% (5/81) suffered from more severe complications, defined as grade II and III according to the classification published in 2011 by Landriel–Ibañez et al (i.e., complications requiring invasive intervention and those requiring intensive care unit admission).^18^ Considering all complications (both medical and surgical) the overall rate increases to 17.3%, which is still lower compared to that of LIF, thus justifying its utility as a less invasive option for spinal fusion.^19^, ^20^, ^21^ Of note, 56.3% of the patients of this cohort had a preoperative ASA score of 3 (defined as an individual with severe systemic disease) and the rate of complications (both medical and surgical) was not different among patients with different ASA scores. Therefore, we conclude that this technique can be safely applied to individuals with a higher frailty index. Nevertheless, due to the potential for facet process rupture during the implantation of this device, we excluded from the study all patients with osteoporosis which in our opinion represents a contraindication to its use.

An important observation that needs to be emphasized is that, within the subset of patients (*n* = 16) undergoing long-term postoperative CT scans analysis, only 56.2% displayed radiologic signs of adequate articular fusion. This rate is notably lower when compared to the combined success rates of instrumented fusion—84.7% for PLF and 94.3% for LIF.^22^ However, caution is warranted in interpreting these findings, considering that the majority of these CT scans (68.7%) were conducted for lumbar spine indications, with a minority for other reasons (e.g., abdominal problems). This discrepancy introduces the possibility of selection bias affecting these results, given that pseudoarthrosis is recognized as a factor influencing outcomes, despite no significant difference in terms of MCID between patients with and without adequate fusion was observed in this small subset of patients.^23^

Additionally, it is crucial to note that, although fusion rates were not exceptionally high, no evidence of device mobilization was identified in the 16 analyzed CT scans. This suggests that while the device may not consistently induce arthrodesis, its ability to effectively stabilize the joints remains intact.

In literature several authors have reported their results with the use of this novel device in lumbar spinal stenosis. Grasso et al compared 40 patients undergoing posterior lumbar decompression with concomitant use of bilateral single-level FW with 40 controls of subjects who were operated of decompression alone.^14^ At a 1-year follow-up, the Authors reported significantly improved outcomes in terms of ODI, VAS and patient-reported outcomes in the group undergoing articular fusion.

Francaviglia et al described their case series which included 38 patients with lumbar degenerative disease (Meyerding grade I degenerative spondylolisthesis, spinal stenosis and disc herniations) with signs of “microinstability”, defined as a hyperintense signal on a T2-wheighted MRI which facet effusion visualized on a T2-wheighted MRI. They found significant improvements in ODI and VAS for low back pain at 1, 3, 6 and 12 months follow-up, reporting 3 complications (two deep vein thrombosis and one wound infection).^8^

Belykh et al used FW to achieve a circumferential fusion in patients with degenerative lumbar disorders in conjunction with different lumbar interbody fusion approaches (ALIF, TLIF and LLIF), reporting 93.8% success rates (define as either good or excellent outcomes).^7^

Regarding instability in lumbar spinal stenosis, it is important to note that, while no clear guidelines for the use of FW have been developed, recent insights in the pathophysiology of degenerative spondylolisthesis have focused the attention on the abnormal motion in the articular joints (caused by the osteoarthritic changes) as the “primum movens” of degenerative changes.^24^ These modifications include progressive vertebral slip, ligamentum flavum hypertrophy, disc degeneration and accelerated osteoarthritic changes.^14^^,^^25^^,^^26^ In light of this theory, degenerative spondylolisthesis (which in 75% of cases presents as grade Meyerding I), is considered as the final stage of a pathological spectrum of conditions characterized by the presence of unstable lumbar spinal stenosis.^27^ In accordance with the assumption which identifies the facet joints as the first cause of degenerative slip, it is reasonable to try to halt this pathological mechanism by fusing the joints to prevent further degenerative progression.

Similarly, lumbar synovial cysts are also associated with vertebral instability and a growing number of surgeons have advocated fusion for this condition.^28^ Because, as previously stated, evidence exist in literature for which synovial cysts originate from instability, a growing number of surgeons now advocate instrumented fusion associated with excision of the cyst. This strategy is further supported by the potential risk of increasing segmental instability (and therefore of predisposing patients to consequent back pain) by means of decompression alone without arthrodesis.^28^, ^29^, ^30^ We are the first to report a small case series of 8 patients undergoing synovial cyst removal and articular fusion with FW, with successful results in 75% of cases (defined as either Macnab excellent or good outcomes). Nevertheless, it is important to note that, while initially we performed cases of unilateral articular fusion (ipsilateral to the synovial cyst), we have abandoned such strategy since two of our 4 patients treated with unilateral fusion developed degenerative pathologies on the contralateral side, including one synovial cyst and one disc herniation at the same level of the facet fusion. It is likely that unilateral block increased mechanical stress on the contralateral side, accelerating degenerative processes. Although ours represents a small series, we do not recommend unilateral articular fusion and fixation procedures anymore.

No study is available in literature analyzing the effects of FW articular fusion at the adjacent level for the treatment or prevention of ASD in patients operated for posterior lumbar interbody fusion (PLIF) with pedicle screw fixation. ASD is a pathological condition characterized by a wide range of degenerative changes, which usually arise after spinal fusion, mostly affecting the lumbar and cervical spine.^31^ Although its pathophysiology is multifactorial, the increased levels of stress transmitted to the adjacent segment (in most cases the upper one) by the rigid fused construct are thought to play a pivotal role in its development.^31^^,^^32^ Importantly, as most patients undergoing PLIF for degenerative spondylolisthesis already present degenerative changes at the time of surgery at the adjacent levels, the question arises as to what is the best management for this condition.^33^ Although many surgeons still perform posterior decompression with laminectomy to prevent (or treat) ASD, recent evidence has revealed how such approach may often prove to be inappropriate due to the fact that it undermines the integrity of the posterior complex between the vertebrae subject to arthrodesis and the adjacent segment, further compromising the stability of the latter and determining the progression of ASD.^33^, ^34^, ^35^, ^36^ While some authors advocate extending instrumented interbody fusion at the adjacent segment with pedicle screws, we have utilized for the first time the less invasive articular fusion with FW technique to prevent ASD.^37^ Although our experience with this indication is still too limited to draw conclusions, we did not report any revisions due to ASD in this small group of patients. Further data will be obviously needed to confirm our hypothesis.

Overall, articular fusion was associated with a limited number of complications, mostly non-severe (grade I according to Landriel–Ibañez scale). Excluding patients who underwent both PLIF + FW, overall complication rate was 18.07% which includes medical complications and minor surgical ones (e.g., dural tears without persistent CSF leak). The rate of complications requiring an invasive procedure (i.e., revision surgery or external spinal drainage) or admission to the ICU (grade II and III) in patients treated with standalone FW application was 7.9%. Nevertheless, such rate includes 2 cases of long-term complications in the synovial cyst group with unilateral articular fusion, a procedure which, as reported above, may be associated with greater long-term risks and that therefore we have abandoned.

Compared with the other more frequently used posterior approaches PLIF and transforaminal lumbar interbody fusion (TLIF), which have been associated with similar overall complication rates of 25%,articular fusion with FW was associated with a lower morbidity profile.^21^^,^^38^ Moreover only 1.1% suffered from postoperative permanent neurological complications as opposed to the rates of PLIF (1.7%–6.5%) and TLIF (1–3%).^21^^,^^38^ It is important to note that some of the reported complications (e.g., dural tears, radicular deficits) are not related to the articular fusion technique by itself (which does not require neural manipulation) but to other surgical maneuvers used to treat associated spinal conditions (e.g., spinal or foraminal decompression, microdiscectomies). Concerning hardware failure, we were able to identify only one case (1.1%) of device mobilization, while frequencies of hardware failure described in literature for PLIF range between 2 and 12%.^39^, ^40^, ^41^ Its simple and geometrically effective design provided with diagonal screws and its facility of insertion in the articular process are likely to provide a significant stability to the FW's construct. Lastly, only 3.4% patients with standalone articular fusion with FW suffered from ASD which required reoperations. Likewise, such rate is also lower to the one reported by a recent meta-analysis by Ren et al in 2018, who reported a mean rate of ASD of 7.8% for LIF procedures.^42^ Of note, while ASD is a time-dependent complication, the minor degree of primary rigidity yielded by articular fusion may modulate mechanical stress on the adjacent segments, resulting in a decreased risk of developing ASD.^31^ In support of this theory, some studies have demonstrated that posterolateral fusion seems to lower the rates of ASD compared to LIF.^31^^,^^43^ Considering that articular fusion with FW shares some important biomechanical similarities with PLF, it may likewise decrease the rates of ASD.^31^^,^^43^

An important comparison with another recent technique of less invasive posterior spinal fusion needs to be conducted with the Midline Lumbar Interbody Fusion (MidLIF).^44^ By employing cortical bone pedicle screws with a medial-to-lateral trajectory, this technique potentially offers an improvement compared to traditional cancellous trajectories. This potential improvement arises from its ability to reduce the necessity for lateral dissection, minimize the risk of postoperative radicular deficits, and facilitate the utilization of screws in osteoporotic patients. A recent meta-analysis has shown a reduction in the rate of complications, surgical duration, blood loss, postoperative ODI, and Japanese Orthopaedic Association (JOA) scores with this technique compared to traditional pedicle screws.^45^ While these encouraging findings may hint at the potential replacement of cancellous screws in the future (particularly in LIF procedures requiring minor degrees of lateral dissection), we posit that FW could still emerge as a minimally-invasive alternative to posterior lumbar fusion (PLF).

Lastly, it is also interesting to observe that patients undergoing multilevel fusions (*n* = 8) did not farecomparably worse with regards to complications and clinical outcomes scores to patients undergoing single level articular fusion. We believe that this finding is also relevant because it adds a therapeutic option to the armamentarium of the spinal surgeon in selected cases of multilevel lumbar degenerative instability in which a multilevel interbody fusion might be considered as “too much”.

## 5 Limitations

Although with 96 cases our study is the largest series in literature describing the use of FW and moreover the one with the longest follow-up (3 years and 9 months), nevertheless some important limitations have to be acknowledged. First of all, it lacks a control group to compare the functional outcomes and rates of complications with other surgical techniques (e.g., LIF, PLF, decompression alone). Secondly, it is difficult to determine what degree (if any) of clinical benefit in our cohort was related to the use of the FW versus other interventions (e.g., laminectomies, foraminotomies, discectomies) which were carried out concomitantly on a case-by-case basis. Thirdly, as previously noted, although this article represents the first attempt to analyze the articular fusion rate with FW (and, moreover, used as a standalone fusion device), it is important to acknowledge the presence of potential selection bias in the analysis. Lastly, in line with the aforementioned theories which identify articular instability as the common first cause of lumbar degenerative disease, we analyzed both degenerative spondylolisthesis and lumbar degenerative disease as a single group. However, a stratified analysis based on the degree of preoperative instability could have shown distinct outcomes between groups.

## 6 Conclusion

In our study, the utilization of the innovative facet wedge system for articular lumbar fixation and fusion seemed to be a secure and efficient approach for achieving lumbar stabilization. Its ease of insertion and its more favorable morbidity profile compared to PLIF and TLIF approaches, make it an attractive method to treat lumbar instability in cases of Meyerding grade I degenerative spondylolisthesis/unstable lumbar stenosis, synovial cysts and for the treatment or prevention of ASD. Furthermore, results and complication rates showed no significant correlation with clinical parameters such as age, ASA score, and BMI, suggesting a favorable profile, even for frail individuals. Future studies will be needed to confirm the low rate of ASD reported in our cohort with the use of standalone FW (3.4%) and to test its utility in the treatment/prevention of ASD in patients treated with LIF.

## Funding

This paper received no specific grant from any funding agency in the public, commercial, or not-for-profit sectors.

## Ethical Compliance Statement

We confirm that we have read the Journal's position on issues involved in ethical publication and affirm that this work is consistent with those guidelines.

## Consent

The patients gave written consent for participating in the procedure, surgical video and publication of their images.

## Data Availability Statement

The data that support the findings of this report are available from the corresponding author upon reasonable request.

## CRediT authorship contribution statement

**Guglielmo Iess:** Writing – original draft, Methodology, Investigation, Formal analysis, Data curation, Conceptualization. **Daniel Levi:** Methodology, Investigation, Data curation. **Raul Della Valle:** Software, Methodology, Data curation. **Giulio Bonomo:** Writing – review & editing, Validation, Investigation. **Giovanni Broggi:** Writing – review & editing, Validation, Supervision. **Marcello Egidi:** Writing – review & editing, Validation, Supervision.

## Declaration of competing interest

The authors declare that they have no known competing financial interests or personal relationships that could have appeared to influence the work reported in this paper.
